# Integrative modeling of diverse protein-peptide systems using CABS-dock

**DOI:** 10.1371/journal.pcbi.1011275

**Published:** 2023-07-05

**Authors:** Wojciech Puławski, Andrzej Koliński, Michał Koliński

**Affiliations:** 1 Bioinformatics Laboratory, Mossakowski Medical Research Institute, Polish Academy of Sciences, Warsaw, Poland; 2 Faculty of Chemistry, University of Warsaw, Warsaw, Poland; Danish Cancer Society Research Center, DENMARK

## Abstract

The CABS model can be applied to a wide range of protein-protein and protein-peptide molecular modeling tasks, such as simulating folding pathways, predicting structures, docking, and analyzing the structural dynamics of molecular complexes. In this work, we use the CABS-dock tool in two diverse modeling tasks: 1) predicting the structures of amyloid protofilaments and 2) identifying cleavage sites in the peptide substrates of proteolytic enzymes. In the first case, simulations of the simultaneous docking of amyloidogenic peptides indicated that the CABS model can accurately predict the structures of amyloid protofilaments which have an in-register parallel architecture. Scoring based on a combination of symmetry criteria and estimated interaction energy values for bound monomers enables the identification of protofilament models that closely match their experimental structures for 5 out of 6 analyzed systems. For the second task, it has been shown that CABS-dock coarse-grained docking simulations can be used to identify the positions of cleavage sites in the peptide substrates of proteolytic enzymes. The cleavage site position was correctly identified for 12 out of 15 analyzed peptides. When combined with sequence-based methods, these docking simulations may lead to an efficient way of predicting cleavage sites in degraded proteins. The method also provides the atomic structures of enzyme-substrate complexes, which can give insights into enzyme-substrate interactions that are crucial for the design of new potent inhibitors.

## Author summary

In recent years, significant progress has been made in the development of experimental and computational methods that predict the structure of biomolecules. As a result, we now have access to an increasing number of high-quality protein structures, which allows us to study not only the functions and mechanisms of individual proteins, but also their complexes or larger aggregates. Therefore, there is an increasing need for new computational tools to investigate larger molecular systems. To effectively model these systems, it is essential to use their simplified representation. In our research, we use the CABS model which utilizes a simplified representation of protein chains to explore two diverse biological processes: the assembly of amyloid protofilaments and the formation of enzyme-substrate complexes. We show that the proposed integrative modeling protocols are capable of predicting the structures of five protofilaments with good accuracy. Furthermore, by relying on the best-scored substrate-enzyme complexes obtained using docking simulations, we are able to identify the positions of the cleavage sites in the peptide substrates of three different proteolytic enzymes. Our results are important for theoretical studies that employ docking strategies, as they can provide valuable insights for scientists carrying out experimental research.

## Introduction

Owing to the immense progress in the sequencing of biological data our knowledge about the chemical composition of biomacromolecules has significantly expanded. Structure description of biomolecules (especially proteins and their complexes) remains more challenging, more costly, and more time-consuming. Therefore, the number of experimentally solved protein structures, while rapidly increasing, remains far beyond the size of the available sequence databases. Since evolutionary changes of protein sequences occurred much faster than their structural differentiations for a significant fraction of newly determined protein sequences, their three-dimensional structures can be predicted computationally using advanced methods of bioinformatics and comparative (homology-based) modeling tools. Very recently, significant progress in protein structure prediction has been achieved using the AlphaFold artificial intelligence system [1]. Therefore, the quantity of known protein structures is now much larger than it was a few years ago. Meanwhile, the number of well-described protein-protein (or protein-peptide) complexes has been increasing much more slowly. Furthermore, studying the processes involved in the pathological states of proteins, e.g. misfolded protein aggregates related to amyloidosis, is very difficult with methods that are deeply dependent on the native state of proteins [2–4]. All-atom molecular dynamics (MD) has proved to be a valuable tool in studying intrinsically disordered proteins and the early steps of amyloid aggregate formation. However, the challenges faced by all-atom MD studies are still significant and numerous [5–9]. Coarse-grained (CG) simulations seem to fill the gap: specifically, parameterized force fields such as MARTINI, AWSEM, and PRIME20 have been successful in many areas, including the structure prediction of simple protein fibrils, their energy landscapes, fibril interaction with bilayers, and prediction of Aβ16–22 solubility [10–14]. There is an urgent need for flexible docking tools which efficiently exploit the plethora of already collected data and address the existing and emerging challenges of structural computational biology [15–18].

Recently a CABS model of protein structure and dynamics was used for designing versatile docking procedures [19–23]. CABS-dock is available as an easy-to-use web server [24] and its standalone version [25] can be freely downloaded by more demanding users. Unlike other recently developed tools for protein-peptide docking CABS-dock enables fully flexible and free docking of peptides, small intrinsically disordered proteins (adapting a specific three-dimensional shape upon the docking), and docking of protein fragments to flexible protein receptors. The receptor structure is treated as a flexible object to accommodate even large modifications of its structure upon docking. The versatility of the CABS-dock program ensures its straightforward connection with experimental or bioinformatic data in easily designed integrative modeling schemes [20]. CABS-dock uses a CG docking simulation. Clustering and MD refinements of the CG models lead to more accurate docking models [26]. It is also worth noting that CABS-based simulations generated using Monte Carlo (MC) sampling scheme, provide not only the structural data, but also overall pictures of the system dynamics and the probable docking pathways [27]. Since the MC trajectories are generated by long series of local moves executed at randomly selected locations, and accepted based on appropriate Boltzmann criteria, the MC simulations mimic long-time snapshots of MD simulations of analogous objects. This has been demonstrated in previous studies using the CABS-based approach, such as studies of folding pathways, and analyses of protein flexibility [28–33]. However, it should be noted that such CG MC simulations lack a pre-defined timescale. The real time-units of the CG MC trajectories can be estimated by comparison with reference MD simulations of equivalent all-atom models.

In this work, drawing from the versatility windows of the original CABS-dock model, we propose two novel integrative modeling protocols and apply them to the molecular description of two important classes of targets from the borderland of structural biology and biomedicine.

In the first of the two proposed protocols, we use fragmentary experimental data and fully flexible docking features of CABS-dock to simulate the assembly of amyloid protofilaments. Amyloid fibrils are long, thin fibers formed by the self-assembly of aggregating proteins or peptides. Such molecular aggregates may cause a wide variety of effects within living cells, in some cases leading to severe diseases, including neurodegenerative and metabolic diseases [34–36]. Protofilaments are smaller, rod-like structures. They are thought to be the precursors to amyloid fibrils, which are thicker, more organized structures made up of multiple protofilaments. Both amyloid fibrils and protofilaments are characterized by high β-sheet content and can adopt different architectures and exhibit different symmetry patterns [37]. Here, a novel application of the CABS-dock tool for modeling protofilament structures formed by multiple in-register copies of parallel peptide chains is demonstrated.

The second protocol is based on the free docking of fully flexible substrates to semi-flexible proteases and is aimed at the identification of the position of enzymatic cleavage sites in peptide substrates. Proteases are enzymes that are responsible for the degradation of proteins and peptides during a process called proteolysis [38,39]. These enzymes can selectively break peptide bonds between particular amino acid residues. This event plays a major role in various cellular processes [40–43]. In the proposed method, we identify the cleaved peptide bond through the analysis of a large number of enzyme-substrate complex structures generated during free docking simulations. This approach differs significantly from the currently used methods based on substrate sequence analysis or machine learning techniques [44]. The obtained modeling results are carefully evaluated and we discuss their implications for a deeper understanding of related biological processes.

## Materials and methods

The CABS-dock, based on CG simulations, is a modeling tool designed for flexible docking of peptides to proteins. The name of the CABS-dock and related CABS modeling tools originates from the pseudo-atoms (C-Alpha, Beta-carbon, and the remaining portion of a Sidechain, if applicable) used for the simplified representation of polypeptide chains. There is one more pseudo-atom, placed in the middle of the distance between two alpha carbons connected by the peptide bond. A similar level of coarse-graining is employed in a few other intermediate resolution models—for example in the UNRES (United Residues) model [45], although other features of apparently equivalent models differ significantly. A conformational state of the CABS polypeptide chain is fully defined by the geometry of its Cα traces, with locations of these pseudo-atoms assigned to the nearest nodes of the underlying cubic-lattice (see ref. [46] for more details). The lattice grid spacing is sufficiently small to ensure that fluctuations in Cα-Cα distances do not impair model resolution. Lattice spacing is also sufficiently low to prevent any noticeable directional biases, typical for more regular lattice models. Main chain Cα traces define the positions of the remaining pseudo-atoms. These are pre-computed (and stored in large data tables) using the local geometry of the main chain defined by the indexes of two adjacent vectors of the Cα trace. Cβ positions are defined by the geometry of natural amino acids, while centers of the remaining portions of the side chains are defined by the statistics of known protein structures. This way only the most probable geometries of the side chains of specific residues are represented. This may look like a drastic simplification, but it is less acute than it may appear. Small fluctuations of main chain distances (and angles) may lead to quite a significant displacement of the side chain and consequently allow better packing of these slightly distorted side chains. In addition, knowledge-based statistical potentials of the CABS force field feature flat and relatively wide minima that allow local adjustments of CG structures without significantly weakening model fidelity.

The force field characterizing CABS proteins is entirely of knowledge-based statistical nature. It includes short-range (along the chain) angular and rotational preferences, enhanced (when available) by predicted (or assigned) secondary structure, assuming the three-letter code convention. To maintain compatibility with the discrete geometry of model chains, the energy values associated with local geometry and residue identities are also to a large extent tabularized, allowing fast computations of energy changes during a simulation process. Long-range interactions (between pseudo-atoms spaced far away along the protein chain) account for the hard excluded volume of the pseudo-atoms, except the united atoms representing the centers of side chains, which are treated as softly repulsive objects. There is also a contact potential for side chains, which depends on the nature of contacting amino acids and the angle of contact. The last factor accounts for an implicit polar solvent, and oppositely charged residues on the protein globule (only parallel or quasi-parallel contacts) are treated as strongly attractive, while the contacts of the same oppositely charged residues in the hydrophobic interior of a globule are very rare, and therefore they are treated as repulsive. Finally, an ersatz of main chain hydrogen bonds is designed, as strong directional interactions between the pseudo-atoms centered on Cα-Cα pseudo-bonds. The hydrogen bonds within a chain fragment showing a helix-like geometry are treated in a slightly different manner. Such a model of interactions reproduces the main structural regularities seen in globular proteins. The fine-tuning of such a force field required careful weighting of various potentials. In particular, the scaling of potentials aimed to realistically reproduce the folding pathways of small proteins. The potential needs to be re-derived from time to time, taking into account the growth of the structural database. Interestingly, these updates did not introduce any significant modifications to the original force fields. This strongly suggests that the model of interactions accounts reasonably for the major regularities observed in globular proteins and enforced by atomistic interactions. Of course, the proposed force field has some limitations. First of all, it is designed only for natural proteins or peptides, and treats the surrounding solvent in an implicit and averaged fashion. For instance, modeling membrane proteins requires significant, although easy to design, modifications of contact potentials [33]. In addition, interactions between different polypeptide chains need to be treated differently.

Conformational space in the CABS model is sampled based on the concept of Monte Carlo Dynamics (MCD). A single time-step of MCD of a polypeptide chain corresponds to a large number (proportional to the chain length) of attempts at small random modifications of the chain shape at randomly selected positions. A variety of local moves are attempted and accepted, or rejected, according to Metropolis’ criterion. The design of these local moves (and the excluded volume of the main-chain pseudo-atoms) prevents self-crossing of the protein chains. This way, the sampling procedure excludes the possibility of artificially formed chain knots. For a very short time related to very local structure shifts, such a model of system evolution is rather vague. However, for broader time scales, the emerging picture of the evolution of modeled systems becomes quite realistic. The MCD of CABS enables not only structure prediction [47,48] but also analysis of folding pathways, local flexibility of protein structures [31,32,49] including structural studies of SARS-CoV-2 spike proteins (see for instance: [50]) or mechanisms of peptide ligand docking. Of course an a priori definition of the correspondence of MCD time steps to real-time units is rather unrealistic. A reasonable MCD time measure can be obtained from experimental measurements of the time (or frequency) of larger structural changes or by comparison with more realistic medium-time MD atomistic simulations of analogous systems [51].

The CABS-dock algorithm [23,25] is a simple extension of the original CABS model. It uses the same discrete representations of polypeptide chains and the same model of interactions and dynamics. There is however a single important modification of the contact potentials that describe interactions between the side chains of different molecules (for example: to a receptor protein and a peptide ligand). The basic version of CABS-dock (server [24]) and its default docking parameters assume a known starting conformation of the receptor protein and randomly distributed ligand molecules around the receptor. The receptor is treated as a flexible object, although its movement is restricted to a defined vicinity of the provided known reference structure. Peptide ligands are treated as fully flexible, without any a priori knowledge of their docking poses and docking sites. Only the amino acid sequence needs to be provided. The default version of CABS-dock uses replica-exchange MC sampling with ten (or twenty) copies of the ligand molecules. The CABS-dock algorithms are very versatile and open to various concepts of integrative modeling [52]. For instance, the user can impose weak (or stronger) biases onto preferred ligand structures (or their fragments). In addition, it is easy to allow full and unrestricted flexibility of selected fragments of the receptor. Many other specific applications of the standalone CABS-dock software are possible. Two new important but not obvious protocols are described in this work.

### Prediction of protofilament structures

CABS-dock standalone [25] version 0.9.18 was used for the test prediction of six distinct protofilament structures composed of in-register parallel peptide chains. The following experimental structures were extracted from the Protein Data Bank database, PDB IDs: 1) 2E8D: a beta2-microglobulin fragment probed by solid-state NMR [53], 2) 2NNT: a structural model of the CA150.WW2 protofilament constructed based on magic-angle-spinning (MAS) NMR spectroscopy, alanine scanning and EM experiments [54], 3) 6TI5: a structural model of Aβ40 fibrils derived from solid-state NMR (SSNMR) experiments [55,56], 4) 6ZRQ: a protofilament amyloid structure of the S20G variant of human amylin (IAPP, islet amyloid polypeptide) solved using cryo-electron microscopy (cryo-EM) [57], 5) 7YAT: a protofilament structure of the hamster prion peptide (sHaPrP, sequence 108–144) solved by cryogenic electron microscopy [58] and 6) 7Q66: a shorter protofilament fragment of the FG-repeat domain of human nucleoporin 98 (Nup98) obtained using a combination of NMR spectroscopy and cryo-electron microscopy [59]. For a detailed description of experimental structures see **Table 1**.

**Table 1:** Experimental structures of protofilaments used for test predictions.

PDB ID	Number of chains	Number of residues in a single chain	Amino acid sequence of a single chain
2E8D	4	22	SNFLNCYVSGFHPSDIEVDLLK
2NNT	4	31	MGATAVSEWTEYKTADGKTFYYNNRTLESTW
6TI5	6	30	EVHHQKLVFFAEDVGSNKGAIIGLMVGGVV
6ZRQ	8	23	FLVHSGNNFGAILSSTNVGSNTY
7YAT	6	25	GAVVGGLGGYMLGSAMSRPMMHFGN
7Q66	11	25	TGTANTLFGTASTGTSLFSSQNNAF

**Fig 1:**
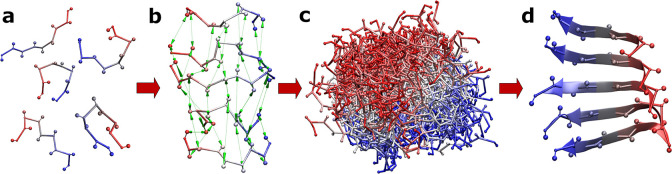
CG docking scheme used for predicting protofilament structures. a) Structures of five copies of a single peptide chain are generated with random conformation and localization. b) Distance restraints imposed on SC atom pairs of the corresponding amino acid residues in adjacent peptide monomers are generated. The restraint distance is set to 5 Å (indicated by green arrows). c) A large number of models are generated during the docking simulation. d) The best models are selected based on the estimated monomer-monomer interaction energies and the values of calculated pcaRMSD parameters. The resulting structures are reconstructed to all-atom representation.

The predicted protofilament structures were generated during a docking simulation followed by a scoring procedure and selection of the best models (the CG docking and scoring scheme used for predicting protofilament structures is shown in **Fig 1**). During each docking simulation, five identical peptide monomers (copies of a single peptide chain) were simultaneously docked to each other. According to the CABS-dock input convention, the first peptide chain was treated as a receptor molecule and the four other monomer chains were treated as docked peptide ligands. Weak distance restraints were imposed between the side-chain (SC) atom pairs of the corresponding amino acid residues in adjacent peptide monomers in the predicted protofilament model. The restraint distance was set to 5 Å which corresponded to the average distance observed in the available protofilament experimental structures. The docking simulation started with a random position and random conformation of each peptide chain. All peptides were fully flexible during the docking calculations. Each simulation was performed using the same CABS-dock input parameters except for the amino acid sequence of each peptide (see supplementary materials, **S1 Table**). During a single docking simulation, 10,000 protofilament models were created and 1,000 lowest energy structures were selected. For each protofilament system, 40 independent docking simulations were completed, resulting in a set of 40,000 low-energy structures.

The selection of the best models was completed according to peptide interaction energy values estimated using CABS statistical potentials or the peptide-chain-average-RMSD (pcaRMSD) parameter or a combination of both. The pcaRMSD value was calculated for each predicted protofilament model using a set of RMSD values obtained during the mutual comparison of all five monomer chains (after structural fitting of two peptide chains for every pair). In this way, the conformations of the five peptides were compared with each other in a given protofilament model. In our earlier work, we showed that low pcaRMSD values correlate with a high level of translational symmetry of monomers within the protofilament structure [19]. The pcaRMSD values were calculated for all predicted models using the following formula:

$$pcaRMSD=\frac{1}{M^{2}-M}\sum_{i=1}^{M}\sum_{j=1}^{M}\sqrt{\frac{1}{N}\sum_{k=1}^{N}{\left|X_{k}^{i}-Y_{k}^{j}\right|}^{2}}$$
**Eq 1.** The pcaRMSD formula used for scoring the predicted protofilament models.

where M is the number of peptide chains in the oligomer model, N is the number of Cα atoms, X_k_ is the coordinate vector for the *k* target Cα atom, Y_k_ is the coordinate vector for the *k* reference Cα atom, whereas *i* and *j* indicate indexes of a particular pair of compared peptide chains in the oligomer model.

### Identification of enzymatic cleavage sites

**Fig 2:**
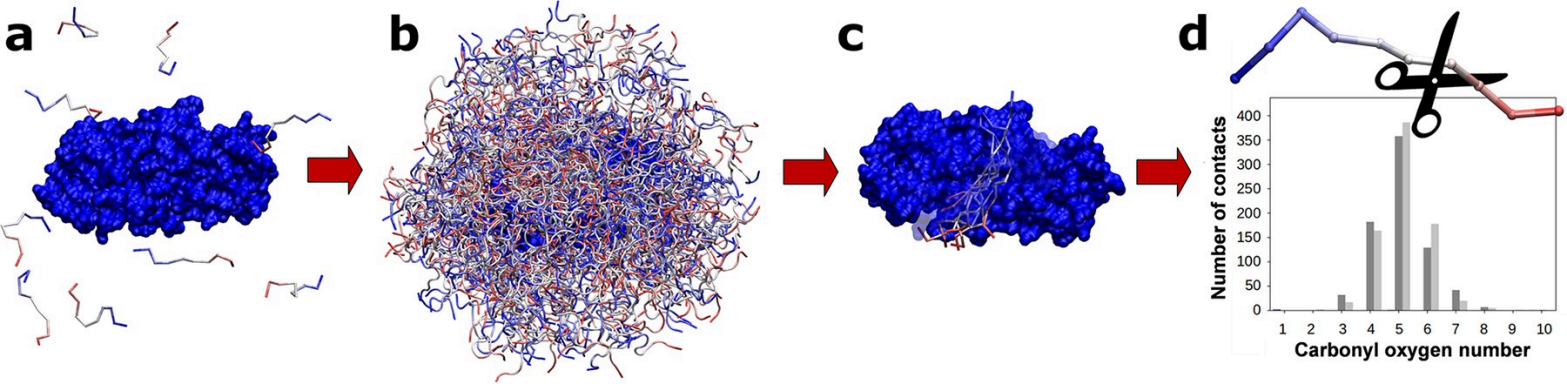
Graphical visualization of the main steps of the procedure used to identify the enzymatic cleavage site on peptide substrates. a) Ten copies of the peptide substrate presenting a random conformation and position are generated. b) A large number of models of enzyme-substrate complexes are predicted during free docking simulations. The peptide ligand is fully flexible and samples the entire surface of the enzyme molecule. c) Hierarchical clustering is used to identify the most probable binding modes of a peptide substrate. Ten representative enzyme-substrate models are selected as central structures of the ten largest clusters identified in the entire model set. d) Histogram analysis of contacts between the substrate and the enzyme active site is used to identify the cleavage site using a large number of enzyme-protein complex models in all-atom representation. The figure shows the results of a single docking simulation of the substrate peptide (sequence: GAETF—YVDGA) to the HIV-1 protease. The enzyme protein is shown in blue surface representation. Peptides are shown in trace representation (peptide residues are colored from red to blue).

**Fig 3:**
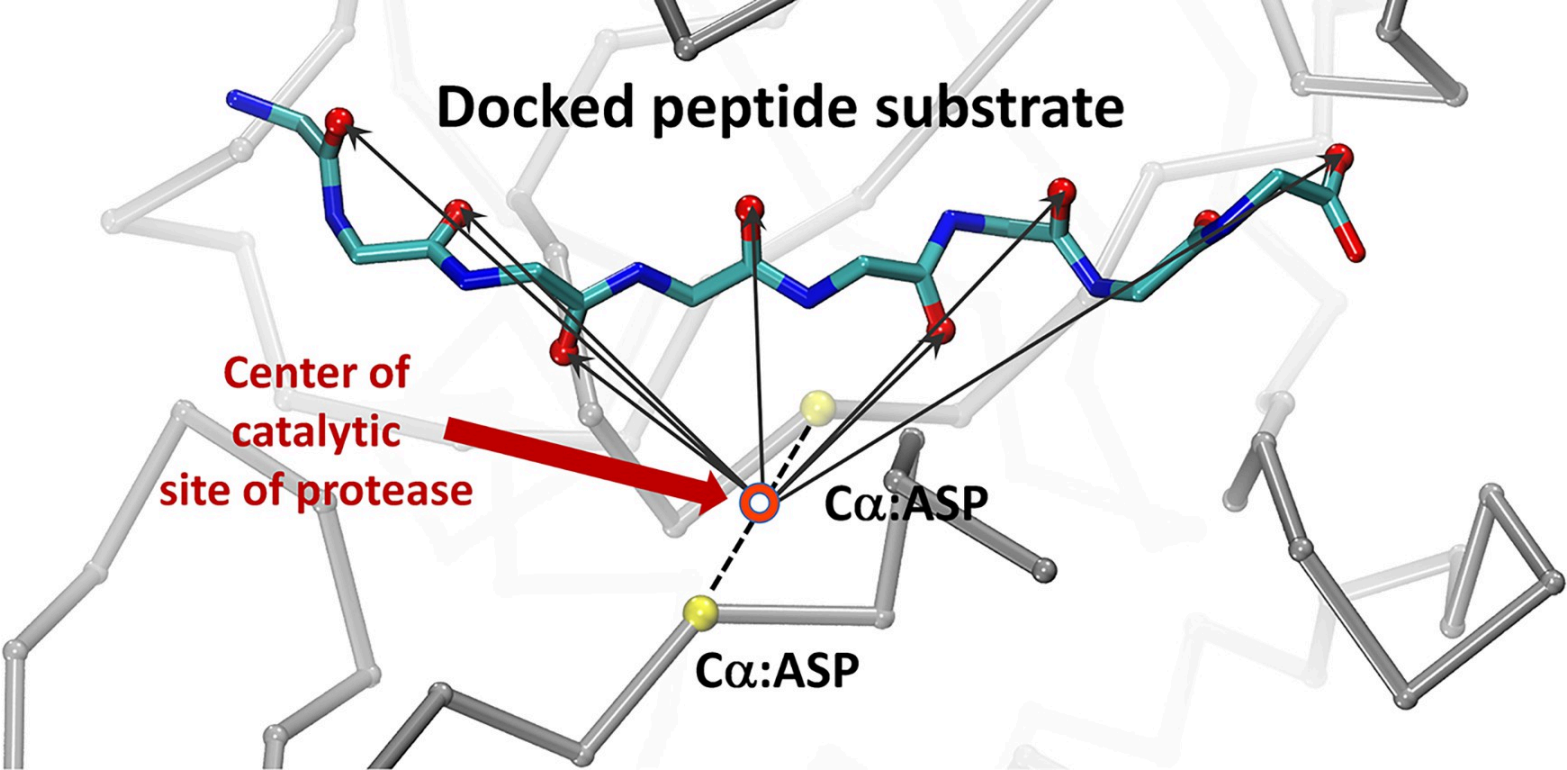
Scheme used to measure distances between the oxygen atoms of the peptide bonds in substrate molecules and the center of the enzyme active site. Black arrows indicate measured distances d_n_. The Cα atoms of the two catalytic Asp residues of the enzyme molecule are highlighted in yellow. The dashed line indicates the line segment connecting the marked Cα atoms. The red circle indicates the center of the active site. Protein fragments of the enzyme are shown using trace representation for clarity.

CABS-dock docking simulations were used to identify cleavage sites in the peptide substrates of proteolytic enzymes. The procedure was similar for all the analyzed systems and included the following steps. First, a large number of docking simulations of a peptide substrate to an enzyme protein were performed without any prior knowledge of the localization of the enzyme’s active site. The peptide ligand was fully flexible and could freely sample the entire surface of the enzyme protein. During a single docking simulation, 10,000 structures of enzyme-substrate complexes were generated. Then, 1,000 models with the lowest protein-peptide interaction energy were selected. Finally, the top 10 models were identified using hierarchical clustering as the central structures of the ten largest clusters. The selected models were reconstructed to all-atom representation using the Modeller program [60]. A large number of docking simulations were completed for each analyzed system. The position of the cleavage site on each peptide was identified based on the analysis of a large number of resulting enzyme-substrate complexes generated using the above procedure (the main stages of the procedure used for the identification of cleavage sites in enzyme substrates are shown in **Fig 2**). For this task, we assumed that the peptide could be cleaved only in the substrate region that directly interacted with the enzyme’s catalytic site. More specifically, the cleavage site was assigned based on the histogram analysis of contacts between the carbonyl oxygen atoms of the peptide bonds in the substrate molecules and the center of the enzyme active site (the peptide bond for which the number of recorded contacts was the highest was considered as a potential cleavage site). Therefore, the center of the active site was approximated by the center of the line segment connecting the Cα atoms of the two catalytic Asp residues of the enzyme molecule. We considered a carbonyl oxygen atom to be in direct contact with the enzyme active site when the measured distance d_n_ between the oxygen atom of the substrate peptide and the center of the enzyme catalytic site was less than or equal to d_n_ ≤ 7 Å. The graphic illustrating the scheme used for distance d_n_ measurements is shown in **Fig 3.**

Fifteen different peptide substrates were analyzed. The first five were docked to a pepsin molecule (PDB ID: 4PEP [61]) and the procedure included 100 independent docking simulations, resulting in 1,000 all-atom models for each enzyme-substrate complex. The same procedure was applied for three substrates of renin protease (using crystal structure PDB ID: 3K1W [62]). The other seven peptides were docked to HIV-1 protease (structure extracted from PDB ID: 3EL1 [63]). In this case, 500 independent docking simulations were conducted resulting in 5,000 enzyme-substrate models for each system. A larger number of docking simulations were completed to account for the possible movement of the two flaps covering the HIV-1 protease active site. Therefore, the two protein fragments (residues 45 to 55 in chain A and chain B) were fully flexible during the docking simulation and no internal restraints were used. For a detailed description of the analyzed systems, see **S2 Table.**

## Results and discussion

### Prediction of amyloid fibril structures

Structures of six different protofilaments with known experimental structures were predicted. The structures were assembled during docking simulations of five interacting peptide monomers. The final protofilament structures were then selected from a large set of protofilament models using structural and energetic criteria. To evaluate the accuracy of this procedure and the quality of the resulting models, we compared the obtained models with their experimental structures.

The standalone version of CABS-dock offers a wide range of adjustable parameters that allow modification of the docking procedure and system properties [20,25]. For instance, applying distance restraints can limit the conformational space of the simulated system, rejecting highly improbable states and allowing more efficient sampling of biologically relevant states. Due to the nature of the modeled system and the structural properties of the protofilaments created by in-register, parallel and identical peptide chains (which resulted in high translation symmetry of peptide chains forming amyloid fibrils) we applied weak distance restraints between the SC united atoms of corresponding amino acid residues in adjacent peptide chains (for a graphical representation of distance restraints, see **Fig 1**, panel **b**). The distance restraints did not determine the resulting shape of the predicted protofilament, but they defined the order of the monomer chains and favored in-register parallel stacking of peptides.

**Fig 4:**
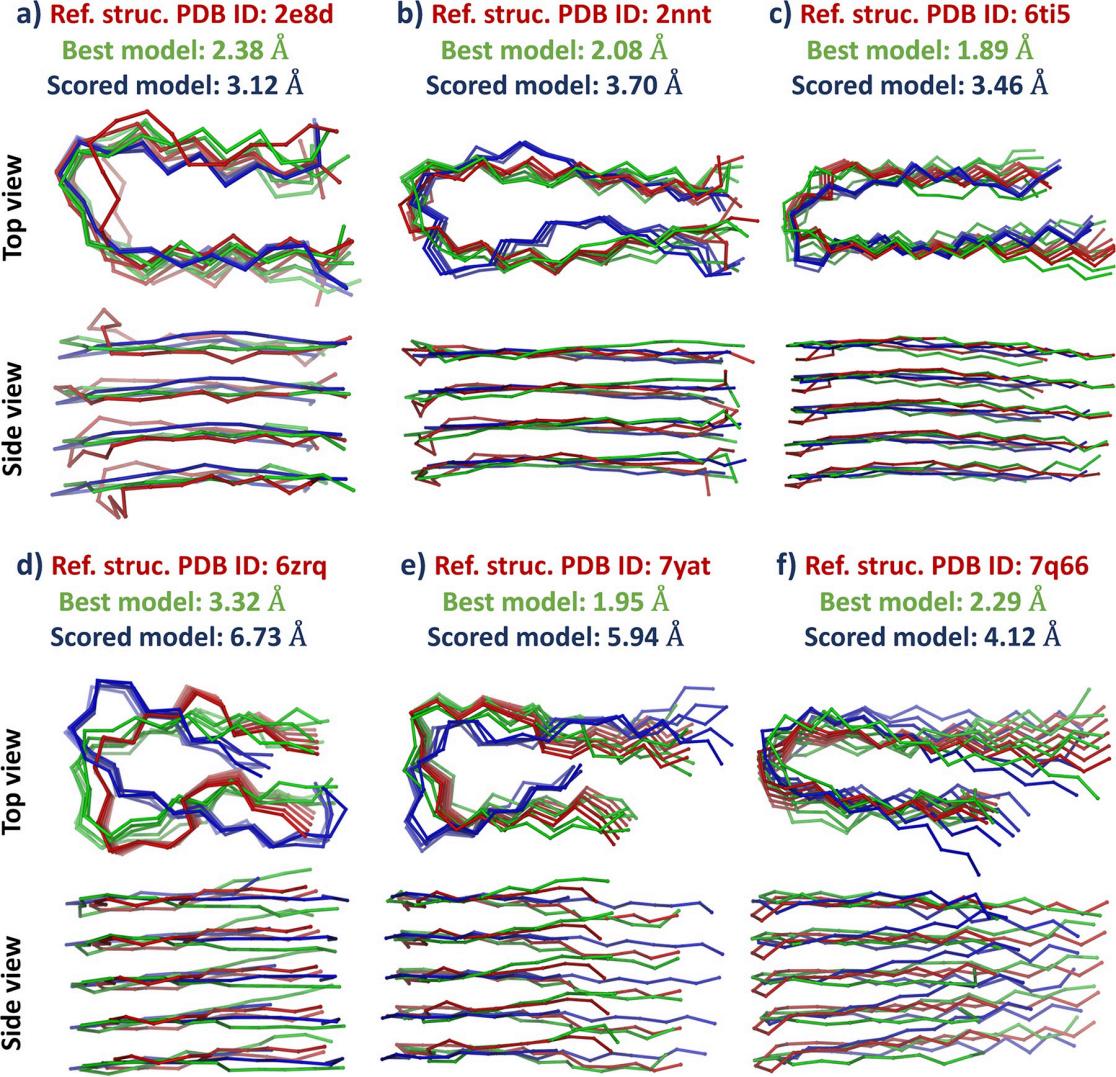
Resulting models of analyzed protofilaments. Experimental structures used as reference structures are shown in red. The best structures (showing the lowest RMSD values) observed in the generated model set (entire docking trajectory) are shown in green. Structures of the best-scored models selected using interaction energy values followed by pcaRMSD calculation are shown in blue. For clarity Cα trace representation is used.

Projecting protein structures onto the lattice used in the CABS model results in a discrete representation of coordinates and allows very fast and efficient sampling of the conformational space. A large number of models can be generated in a relatively short time, which does not require large computing resources. Typically, this large set of models contains models that are very close to their native (or experimentally derived) structures and the most challenging step is to identify the best model. Our results show that among all the predicted models there were protofilament structures with low RMSD values, when compared to their experimental structures, in the range of 1.82 Å to 3.32 Å (see **Table 2**). Those structures were almost identical to the reference structures (see **Fig 4**).

**Table 2:** RMSD values of protofilament models obtained using different scoring methods.

PDB ID:	Best from all^a^ [Å]	Results for different scoring methods [Å]							
		E		pcaRMSD		E_1000_ » pcaRMSD		pcaRMSD_1000_ » E	
		Best scored	Best from top10^b^ scored	Best scored	Best from top10^b^ scored	Best scored^c^	Best from top10^b^ scored	Best scored	Best from top10^b^ scored
**2E8D**	2.38	5.0	3.01	3.15	3.08	3.12	2.86	3.55	2.96
**2NNT**	2.08	4.02	3.10	3.67	2.81	3.70	2.72	3.22	2.71
**6TI5**	1.89	4.26	3.49	3.46	2.96	3.46	2.76	4.26	3.49
**6ZRQ**	3.32	6.24	4.3	7.01	5.21	6.73	4.28	6.54	4.28
**7YAT**	1.95	5.88	4.66	7.16	3.60	5.94	3.73	4.89	3.74
**7Q66**	2.29	5.67	4.11	4.02	3.96	4.12	4.01	4.12	3.40

^a^ The best models (showing the lowest RMSD values) observed in the generated model set (entire docking trajectory). Structures of the best models are shown in **Fig 4** in green.

^b^ The lowest RMSD values observed in the set of top 10 scored models.

^c^ The best scored models using interaction energy values followed by pcaRMSD calculation are shown in **Fig 4** in blue.

**Fig 5:**
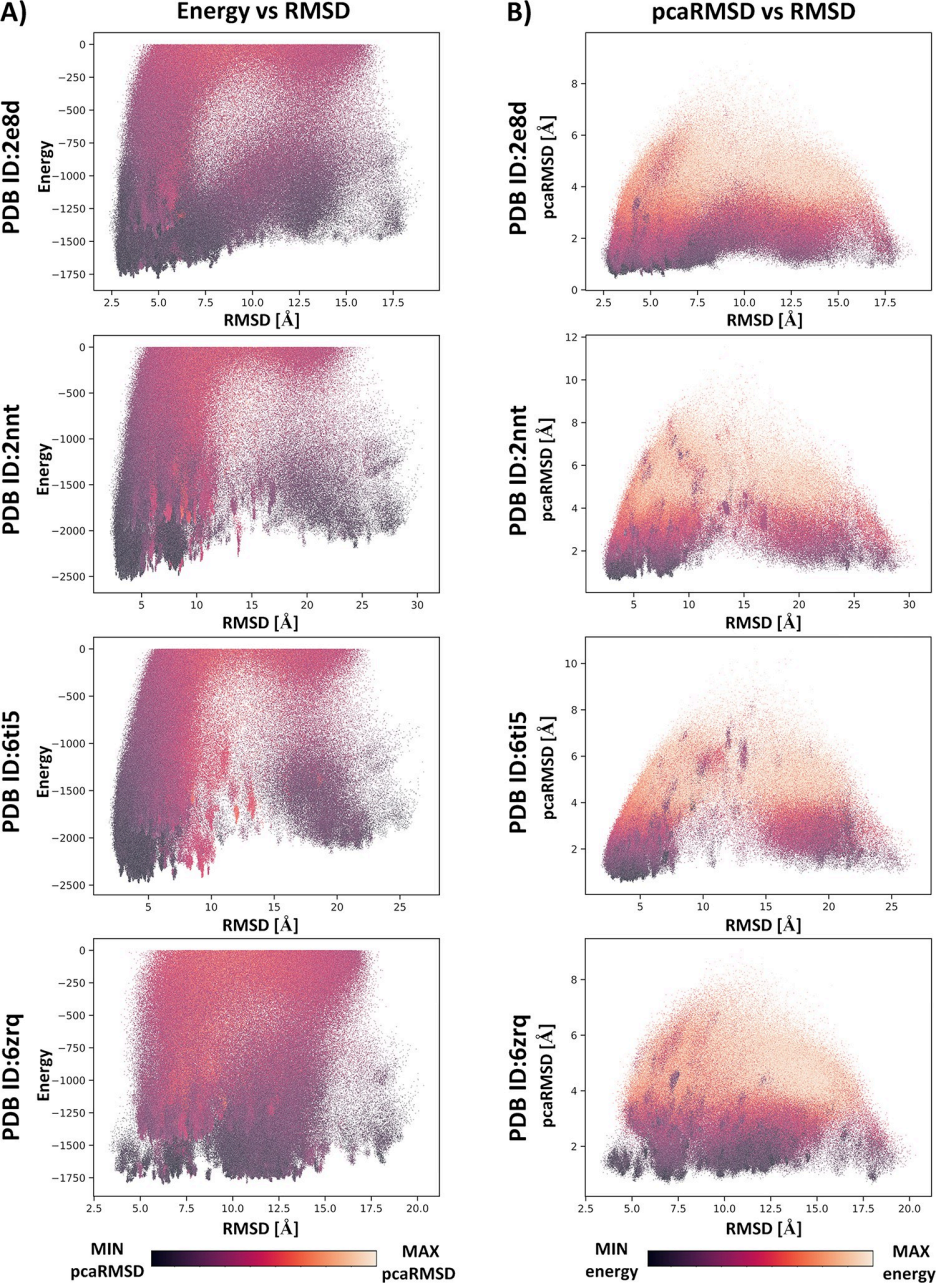
Correlation between interaction energy, pcaRMSD and RMSD for four predicted protofilament models. Plots show the correlation between interaction energy values (panel A) and the pcaRMSD parameter (panel B) and the RMSD values calculated using the experimental structures as a reference for a large number (~40,000) of generated protofilament models. At the bottom of each panel there is a color bar describing the colors used for the different values of estimated interaction energy and the pcaRMSD parameter.

Model scoring and selection of the most accurate structures in the proposed procedure were conducted based on two parameters: the interaction energy values estimated for the bonded monomers and the values of the pcaRMSD parameter, related to the translational symmetry of bonded peptide monomers in the protofilament structure. Scoring based solely on interaction energy values failed to identify high-quality models (see **Table 2**). Better results were obtained by scoring based on the pcaRMSD value, which was consistent with the fact that the structure of the protofilament, with its in-register parallel peptide chain architecture, should show high translational symmetry of interacting peptide monomers. The analysis of the interaction energy and pcaRMSD values calculated for all generated models showed a correlation with model accuracy, as evidenced by the low RMSD values when compared to the reference structure (refer to **Figs 5** and **S1**). On average, the 1,000 models with the lowest energy/pcaRMSD values had an RMSD approximately 3.9 Å lower than the rest of the set, see **S3 Table**. However, it was impossible to identify the single most accurate structure among the entire set of models. To improve the scoring results, we tested a combination of these two parameters in two variants of the scoring procedure. In the first variant, the 1,000 models with the lowest interaction energy values were selected, and then the best model was chosen based on the lowest pcaRMSD value. The second variant of the scoring was similar. First, the 1,000 models with the lowest pcaRMSD values were selected, and then the model with the lowest interaction energy was chosen as the best-predicted structure. The first variant of the procedure proved to be more accurate and identified models with lower RMSD values (based on a comparison to the reference structures). The data for the best-identified structures are shown in **Table 2**, and the predicted structures for six protofilaments are shown in **Fig 4.**

The accuracy of the proposed protocol for predicting protofilament structures can be further increased by applying an MD-based refinement and model scoring procedure. In our recent work, we showed that even short MD simulations of CABS-generated models with reconstructed atomistic details can be efficiently used for model validation [26]. The receptor-peptide interaction energy values estimated during all-atom MD simulations with explicit solvent can be successfully used to identify the most accurate models, which are close to experimentally derived structures [26]. A similar procedure, followed by the analysis of the translational symmetry of monomers in the obtained protofilament models after MD optimization (e.g., using pcaRMSD parameter values), could probably increase prediction accuracy. Other scoring functions or geometry optimization protocols or consensus scoring functions based on the combination of different methods (e.g., Rosetta [64], GalaxyPepDock [65], HADDOCK [66], pepATTRACT [67]) could also improve the quality of the selected models.

Fast and accurate protocols for modeling protofilament structures can be the first step toward designing a method for predicting the structures of whole amyloid fibrils. It has been demonstrated that long fibril structures (formed by amyloidogenic insulin fragments) can be assembled using optimized protofilament structures as building blocks repeated along the long fibril axis [19]. We are currently working on a similar procedure to predict fibrils for other aggregating peptides, and the results will be published soon. The CABS-dock method enables the modification of distance restraints between the desired united atoms (or protein fragments) in a simulated system, expanding the possibilities for modeling different protofilament architectures with different symmetry patterns [37].

### Identifying the positions of enzymatic cleavage sites in peptide substrates

The positions of enzymatic cleavage sites were identified using CABS-dock docking simulations for fifteen peptides: five pepsin substrates consisting of 8 to 21 amino acid residues, three renin substrates consisting of 8 to 18 amino acid residues and seven HIV-1 protease substrates containing of 8 to 16 amino acid residues. The predicted positions of the cleaved peptide bonds were then compared to experimental data from the MEROPS database [68] and published papers [69–74].

**Fig 6:**
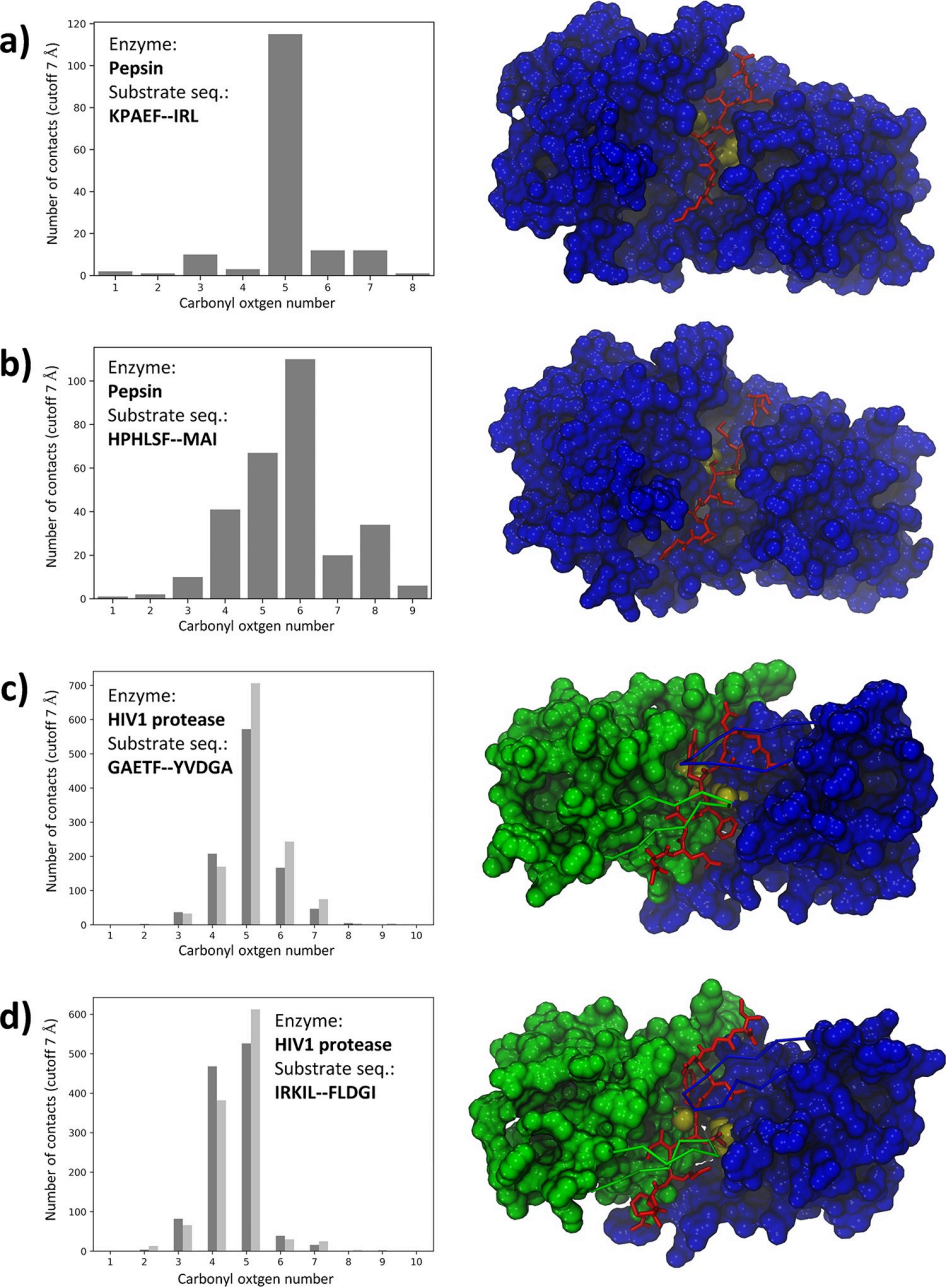
Results of cleavage site prediction for four selected peptide substrates. The position of the cleavage site was identified using histogram analysis of the contacts between the carbonyl oxygen atoms of the substrate peptide bonds and the enzyme’s active site. On the left side of each panel, a bar plot presenting a histogram for the cutoff distance (d_n_ ≤ 7 Å) is shown. On the right side, an example structure of a docked substrate is displayed in a conformation that allows the proteolytic cleavage reaction. The enzyme molecules are shown in a surface representation (pepsin is shown in blue, and two symmetric domains of HIV-1 protease are shown in green and blue, respectively). In the center of the active sites two catalytic AspSP residues are shown in yellow. The two flaps of HIV-1 protease are shown in a trace representation for clarity. The docked substrate is shown in a stick representation and is colored red.

The three selected proteolytic enzymes were aspartate proteases, meaning they had two catalytic aspartate residues in their active sites. The exact location of the cleavage site on each peptide substrate was predicted by analyzing substrate-enzyme contact information from a large number of generated molecular models of enzyme-substrate complexes. Specifically, histogram analysis of all distances measured from the carbonyl oxygen atoms of all the substrate peptide bonds to the center of the enzyme’s active site was performed. According to the enzymatic catalysis mechanism of aspartate proteases, proteolytic cleavage begins with a nucleophilic attack of the catalytic water molecule on the carbonyl carbon atom of the scissile bond [38,39,75]. In this study, we assumed that cleavage occurred at the peptide bond whose carbonyl oxygen atom was most often located near the center of the protease’s active site in the set of predicted complex structures. The results of histogram analysis for the four selected systems are shown as bar plots in **Fig 6**.

The catalytic site of the pepsin molecule lies in the middle of a long cleft located between the two domains of the enzyme molecule (residues 1 to 175 and residues 176 to 327, respectively) [76]. Since the two domains of pepsin are not symmetrical, the local orientation of the substrate peptide chain at the center of the active site during enzymatic degradation must be strictly defined. This fact must be taken into account when predicting enzyme-substrate complexes. In the histogram analysis of pepsin-substrate systems, we included only those models in which the orientation of the substrate peptide chain along the cleft near the active site was similar to that observed in pepsin inhibitor structures (e.g., PDB ID: 1PSA [77]) and was also consistent with our previous studies [21]. In the case of renin-substrate complexes, we followed a similar approach as it has a structure comparable to that of pepsin, including the shape of the catalytic site (PDB ID: 3K1W [78]).

The positions of the cleavage sites identified using histogram analysis for two pepsin substrates KPAEF—IRL and HPHLSF—MAI show excellent agreement with experimental data [69]. For the first system, in 115 predicted models, the carbonyl oxygen atom in the 5^th^ peptide bond was located within the cutoff distance (d_n_ ≤ 7 Å) of the active site (see the left side of the panel (a) of **Fig 6**). The remaining oxygen atoms of other peptide bonds did not tend to stay near the binding site. In the second examined pepsin substrate, the cleavage site was also clearly identified on the 6^th^ peptide bond. The number of structures in which the oxygen atom was within the cutoff distance was 110. For the 5^th^ oxygen atom, the number of structures fulfilling the distance criteria was also high (67 models) but significantly smaller than for the 6^th^ oxygen (see the left side of the panel (b), **Fig 6**). The peptide chains of both ligands preferred to adopt an extended conformation along the cleft surrounding the active site. Two structures showing a ligand-bound conformation that allows cleavage of the accurately identified peptide bond are shown on the right side of panels (a) and (b), **Fig 6.**

For systems, which included HIV-1 protease, we conducted the histogram analysis by considering two different substrate peptide binding modes that were characterized by the opposite direction of the peptide chain extending along the enzyme active site (gray and black bars, **Fig 6**). This was necessary because HIV-1 protease is composed of two identical domains which make the enzyme molecule symmetrical. Therefore the same substrate can bind in the opposite direction of the peptide chain along the cleft between the two enzyme domains, which in turn shows two equivalent binding modes. The cleavage site was identified on the 5^th^ peptide bond of the two analyzed HIV-1 protease substrates GAETF—YVDGA, IRKIL—FLDGI which was positioned in the center of the two peptide chains (see **Fig 6**, panels c) and d)). To assess whether the length of the docked peptide could affect the prediction results of the cleavage site position (which was exactly in the center of the substrate peptide chain), we performed two additional docking simulations using longer variants of the HIV-1 substrate (seq.: GAETF—YVDGA). Since this peptide is part of GAG polyprotein [71], the main structural protein of HIV-1 and all other retroviruses [79], we constructed two longer substrate variants by adding additional amino acid residues to the C- or N-terminus of the substrate peptide chain according to the GAG sequence. The first substrate variant (seq.: TEPISGAETF—YVDGA) was created by adding five residues at the beginning of the peptide chain, while the second substrate variant (seq: GAETF—YVDGAANRET) was extended by adding five residues to the C-terminus. The cleavage sites in the two longer substrate variants were correctly identified using the proposed docking procedure, suggesting that length did not influence prediction accuracy. Bar plots presenting histogram analysis are shown in **S2 Fig**.

**Fig 7:**
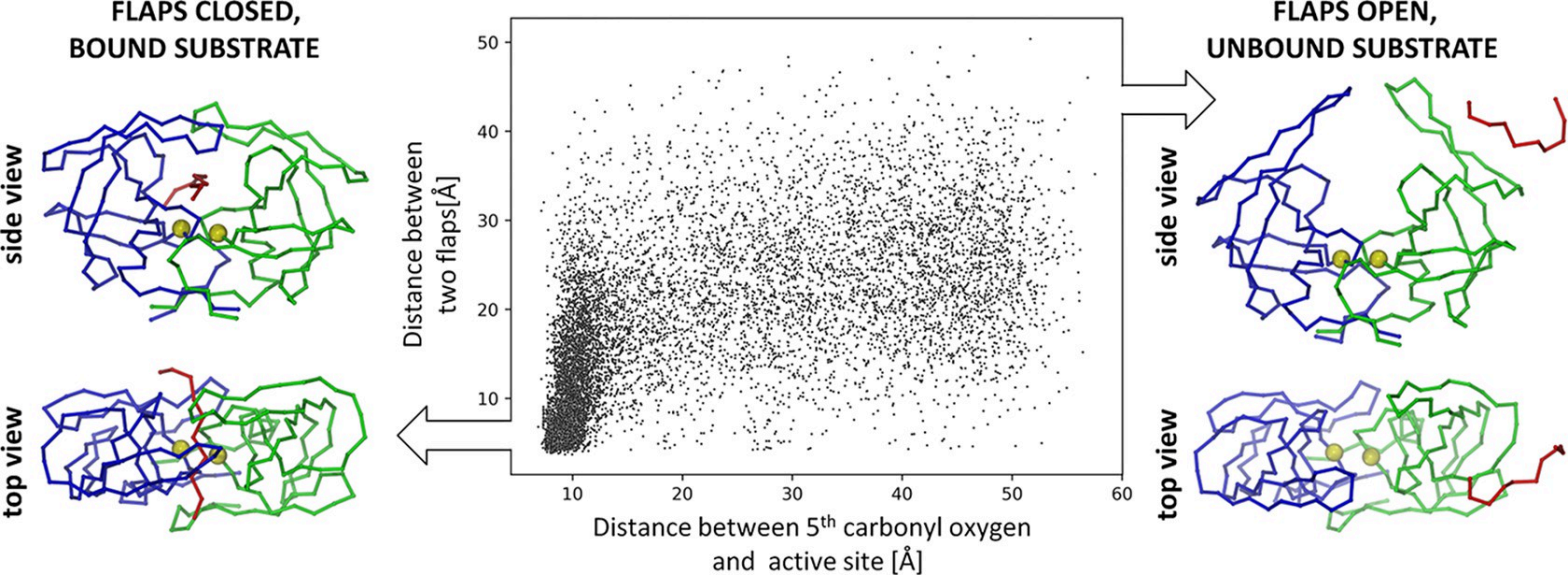
Correlation between substrate binding and movement of the flaps of HIV-1 protease observed during a docking simulation. Ligand binding is characterized by the distance between the carbonyl oxygen atom of the substrate’s 5^th^ peptide bond and the center of the enzyme’s active site (x-axis on the plot). Flap movement is monitored as the distance between the Cα atoms of two GLY:52 residues located in chains A and B of HIV-1 protease (y-axis on the plot). The left panel shows HIV-1 protease with a bound substrate and closed flaps. The right panel shows the enzyme without a bound substrate and with open flaps. The enzyme and substrate are presented in a trace representation for clarity. Two HIV-1 protease domains are shown in blue and green. The substrate is marked in red. Two catalytic aspartic acid residues, showing the center of the enzyme’s active site, are marked with yellow spheres.

The active site of HIV-1 protease is covered by two identical flaps, residues from 45 to 55 of each domain [63]. The flap region is critical for binding substrates or inhibitors and also for catalytic activity of the enzyme [80]. Flap movement has been also reported in numerous works [81–83]. During all docking simulations, the flaps were fully flexible and transitions between open and closed conformations were observed. Interestingly, analysis of the MC trajectory (Cα trace representation only) generated during the docking simulation shows a correlation between the closed state of the two flaps and the ligand binding mode that is optimal for substrate cleavage (**Fig 7**). For bound ligands (with a distance of less than 9 Å between the center of the enzyme catalytic site and the scissile bond in substrate), we observed closed flap conformations in over 91% of cases (with a distance of less than 12 Å between Cα atoms of GLY:52 in chain A and GLY:52 in chain B of enzyme). In contrast, for unbound ligands, closed flap conformations were observed only 25% of the time. This was in agreement with previous findings showing that the flaps need to first open for the peptide to bind and that the protease interaction with the bound substrate influenced the flap opening frequency and interval [84].

The positions of cleavage sites identified for all fifteen enzyme-substrate systems are listed in **Table 3**. For comparison, we have included the prediction results obtained for pepsin and HIV-1 protease using PeptideCutter and HIVcleave servers, respectively. Our method accurately predicted cleavage sites for 12 systems. However, it should be noted that the predictions generated using the servers may overestimate the number of possible cleavage positions, depending on the chosen cutoff threshold value [85]. The servers identified between one and five cleavage sites. The HIVcleave method failed to identify cleaved bonds correctly for two HIV-1 protease substrates (seq.: SYFNLNPFEVL—QIDPE and seq.: NVVNSGGMVM—MVPGAG). To the best of our knowledge, no methods are currently available for the straightforward prediction of cleavage sites on renin peptide substrates.

**Table 3:** Predicted positions of cleavage sites in peptide substrates.

N.o.	Enzyme name	Substrate sequence (“—” cleavage site)	Cleavage site position (peptide bond number)		
			Exp. data^a^	CABS-dock	Servers^b^
1	Pepsin	KPAEF—IRL	5	5	4, 5, 8
2	Pepsin	HPHLSF—MAI	6	6	4, 6
3	Pepsin	AFPLEF—IREL	6	6	2, 3, 5, 6, 9
4	Pepsin	ENESAEAFPLEF—IRELEGER	12	12	8, 9, 11, 12, 15
5	Pepsin	TMARHPHPHLSF—MAIPPKKNQ	12	14	10, 12
6	Renin	PFHL—LVYS	4	6	n.d.
7	Renin	PYIL—KRGS	4	4	n.d.
8	Renin	DRVYIHPFHL—VIHNESTC	10	10	n.d.
9	HIV-1	GAETF—YVDGA	5	5	5
10	HIV-1	IRKIL—FLDGI	5	5	5
11	HIV-1	TEPISGAETF—YVDGA	10	10	5, 6, 7, 10
12	HIV-1	GAETF—YVDGAANRET	5	5	5
13	HIV-1	AAGAVASYDY—LVIGGG	10	9	7, 10, 11, 12
14	HIV-1	SYFNLNPFEVL—QIDPE	11	11	7, 8, 10
15	HIV-1	NVVNSGGMVM—MVPGAG	10	10	5, 6, 7, 9

^a^ Experimental data extracted from literature [68–74].

^b^ Cleavage positions identified using PeptideCutter (pepsin substrates) and HIVcleave (HIV-1 substrates, cutoff threshold R = 0) servers [72,78].

Currently, computational methods used for predicting enzymatic cleavage sites in peptide or protein substrates can be divided into two groups based on their methodologies: sequence scoring methods and machine learning techniques [44]. Sequence scoring methods are generally less accurate, but they can produce results promptly. These methods use scoring functions based on large datasets of experimentally verified cleavage site positions in a large number of analyzed sequences. Predictions are made by comparing the query sequence to these known datasets. On the other hand, machine learning methods are more sophisticated and generally perform better, but they require well-assembled datasets for training, selection of an appropriate training model, and model evaluation and optimization [44]. In addition, machine learning methods can incorporate various heterogeneous features of cleaved substrates, including evolutionary information, physiochemical properties, and structural features during model training, which leads to better prediction accuracy. The proposed procedure for identifying cleavage sites based on docking simulations differs from the typical methods mentioned above. It relies entirely on enzyme-peptide interactions, meaning that the structural features of enzyme molecules and the dynamic character of the peptide ligand are essential for correctly positioning the substrate and allowing proteolytic degradation of a specific peptide bond. This approach is slower and requires more computational resources, but it takes into account all structural and dynamic features of the bound substrate, such as secondary structure preferences, multiple peptide chains, intramolecular disulfide bonds, exposed fragments of the peptide chain, and local substrate unfolding. In our recent work, we used a similar method to identify pepsin cleavage sites on the insulin molecule, and the simulation results were consistent with experimental findings [21]. We were able to correctly identify several cleavage sites on the substrate molecule. It is also worth noting that the molecular structure of insulin includes two peptide chains stabilized by three internal disulfide bonds, which determine its topology and conformation [21]. We are currently developing this method to enable the scanning of longer peptide/protein chains for potential cleavage sites and for the design of potent enzyme inhibitors.

In summary, we propose two new less obvious CABS-dock-based docking protocols. Since the CABS-dock algorithms are very flexible and allow easy-to-apply modifications of their default options, the range of integrative modeling schemes is very broad. The first of the two proposed modeling protocols addresses the early stages of amyloid formation: the assembly of small protofilaments. The test predictions were performed on several oligopeptide complexes whose three-dimensional structures have been determined experimentally. Five copies of peptide forming protofilaments were subject to simultaneous docking simulations, where one of the five peptides was treated as a flexible receptor, while the remaining four peptides were treated as fully flexible ligands. Known experimentally determined common structural regularities of expected protofilaments were imposed in a form of weak distance restraints, favoring parallel, in-register contacts of the peptide chains. The resulting structures after appropriate ranking were of good accuracy, providing structural data for further computational studies of amyloid formation. The second docking protocol was dedicated to predicting the positions of cleavage sites on peptide substrates of proteolytic enzymes. By analyzing many docking poses and specific geometries of the receptor-substrate complexes, those apparently associated with proteolytic degradation were identified in the analyzed systems. It has been shown that this strategy allows accurate identification of the cleaved peptide bonds, which is of great importance for structural biology and molecular medicine. Both protocols presented in this work prove that using a combination of multiscale modeling and known experimental facts, along with properly targeted search regions in efficient docking procedures, provides numerous opportunities for new computational studies of biomolecular complexes.

## Supporting information

S1 Table Example CABS-dock simulation input parameters used in this study. (DOCX)

S2 Table Description of enzyme-substrate systems analyzed in this study. (DOCX)

S3 Table Average RMSD values for the top 1000 models with the lowest energy/pcaRMSD values and for the remaining set of models. The average RMSD values were calculated as the arithmetic mean of each set, while the errors were defined as the standard deviations. The first set of models consisted of the 1,000 structures with the lowest interaction energy (or lowest pcaRMSD values), while the second set consisted of the remaining 39,000 models.
(DOCX)

S1 Fig Correlation between interaction energy, pcaRMSD and RMSD for the two predicted protofilament models. Plots show the correlation between interaction energy values (panel A) and the pcaRMSD parameter (panel B) and the RMSD values calculated using the experimental structures as a reference for a large number (~40,000) of generated protofilament models. At the bottom of each panel there is a color bar describing the colors used for the different values of estimated interaction energy and the pcaRMSD parameter.
(DOCX)

S2 Fig Results of cleavage site prediction for two HIV-1 protease peptide substrates. The position of the cleavage site was identified using histogram analysis of the contacts between the carbonyl oxygen atoms of the substrate peptide bonds and the enzyme’s active site. Bar plots show histograms for the cutoff distance (d_n_ ≤ 7 Å).
(DOCX)

S1 File Predicted structural models of analyzed systems. Files in pdb format containing atomic coordinates of the structures obtained in this study: the highest-scored amyloid models and 10 examples of the docked peptide for each enzyme-substrate complex.
(ZIP)
