# Supplementary material for: Integrative modeling of diverse protein-peptide systems using CABS-dock
Source: PLoS Comput Biol. 2023 Jul 5;19(7):e1011275. doi: 10.1371/journal.pcbi.1011275 (PMC10351741; doi:10.1371/journal.pcbi.1011275)
Supplement: S1 Table — (DOCX) [file pcbi.1011275.s001.docx]

**S1 Table.** **Example CABS-dock simulation input parameters used in this study.**

| **Modeling task** | **Cabs-dock input command** | **Additional parameters** |
| --- | --- | --- |
| Prediction of protofilament structure (model PDBID: 2E8D) | CABSdock -s 100 -M -A -C -S -v 4 --no-protein-restraints -i monomer.pdb -p SNFLNCYVSGFHPSDIEVDLLK:EEEEEEEEEEEEEEEEEEEEEE -p SNFLNCYVSGFHPSDIEVDLLK:EEEEEEEEEEEEEEEEEEEEEE -p SNFLNCYVSGFHPSDIEVDLLK:EEEEEEEEEEEEEEEEEEEEEE -p SNFLNCYVSGFHPSDIEVDLLK:EEEEEEEEEEEEEEEEEEEEEE -p SNFLNCYVSGFHPSDIEVDLLK:EEEEEEEEEEEEEEEEEEEEEE --sc-rest-file sc_restraints >& CABS.log | Distance restraints between monomers, file sc_restraints:  1:A 1:PEP1 5.0 1.0  2:A 2:PEP1 5.0 1.0  3:A 3:PEP1 5.0 1.0  4:A 4:PEP1 5.0 1.0  5:A 5:PEP1 5.0 1.0  6:A 6:PEP1 5.0 1.0  7:A 7:PEP1 5.0 1.0  8:A 8:PEP1 5.0 1.0  9:A 9:PEP1 5.0 1.0  10:A 10:PEP1 5.0 1.0  11:A 11:PEP1 5.0 1.0  12:A 12:PEP1 5.0 1.0  13:A 13:PEP1 5.0 1.0  14:A 14:PEP1 5.0 1.0  15:A 15:PEP1 5.0 1.0  16:A 16:PEP1 5.0 1.0  17:A 17:PEP1 5.0 1.0  18:A 18:PEP1 5.0 1.0  19:A 19:PEP1 5.0 1.0  20:A 20:PEP1 5.0 1.0  21:A 21:PEP1 5.0 1.0  22:A 22:PEP1 5.0 1.0  1:PEP1 1:PEP2 5.0 1.0  2:PEP1 2:PEP2 5.0 1.0  3:PEP1 3:PEP2 5.0 1.0  4:PEP1 4:PEP2 5.0 1.0  5:PEP1 5:PEP2 5.0 1.0  6:PEP1 6:PEP2 5.0 1.0  7:PEP1 7:PEP2 5.0 1.0  8:PEP1 8:PEP2 5.0 1.0  9:PEP1 9:PEP2 5.0 1.0  10:PEP1 10:PEP2 5.0 1.0  11:PEP1 11:PEP2 5.0 1.0  12:PEP1 12:PEP2 5.0 1.0  13:PEP1 13:PEP2 5.0 1.0  14:PEP1 14:PEP2 5.0 1.0  15:PEP1 15:PEP2 5.0 1.0  16:PEP1 16:PEP2 5.0 1.0  17:PEP1 17:PEP2 5.0 1.0  18:PEP1 18:PEP2 5.0 1.0  19:PEP1 19:PEP2 5.0 1.0  20:PEP1 20:PEP2 5.0 1.0  21:PEP1 21:PEP2 5.0 1.0  22:PEP1 22:PEP2 5.0 1.0  1:PEP2 1:PEP3 5.0 1.0  2:PEP2 2:PEP3 5.0 1.0  3:PEP2 3:PEP3 5.0 1.0  4:PEP2 4:PEP3 5.0 1.0  5:PEP2 5:PEP3 5.0 1.0  6:PEP2 6:PEP3 5.0 1.0  7:PEP2 7:PEP3 5.0 1.0  8:PEP2 8:PEP3 5.0 1.0  9:PEP2 9:PEP3 5.0 1.0  10:PEP2 10:PEP3 5.0 1.0  11:PEP2 11:PEP3 5.0 1.0  12:PEP2 12:PEP3 5.0 1.0  13:PEP2 13:PEP3 5.0 1.0  14:PEP2 14:PEP3 5.0 1.0  15:PEP2 15:PEP3 5.0 1.0  16:PEP2 16:PEP3 5.0 1.0  17:PEP2 17:PEP3 5.0 1.0  18:PEP2 18:PEP3 5.0 1.0  19:PEP2 19:PEP3 5.0 1.0  20:PEP2 20:PEP3 5.0 1.0  21:PEP2 21:PEP3 5.0 1.0  22:PEP2 22:PEP3 5.0 1.0  1:PEP3 1:PEP4 5.0 1.0  2:PEP3 2:PEP4 5.0 1.0  3:PEP3 3:PEP4 5.0 1.0  4:PEP3 4:PEP4 5.0 1.0  5:PEP3 5:PEP4 5.0 1.0  6:PEP3 6:PEP4 5.0 1.0  7:PEP3 7:PEP4 5.0 1.0  8:PEP3 8:PEP4 5.0 1.0  9:PEP3 9:PEP4 5.0 1.0  10:PEP3 10:PEP4 5.0 1.0  11:PEP3 11:PEP4 5.0 1.0  12:PEP3 12:PEP4 5.0 1.0  13:PEP3 13:PEP4 5.0 1.0  14:PEP3 14:PEP4 5.0 1.0  15:PEP3 15:PEP4 5.0 1.0  16:PEP3 16:PEP4 5.0 1.0  17:PEP3 17:PEP4 5.0 1.0  18:PEP3 18:PEP4 5.0 1.0  19:PEP3 19:PEP4 5.0 1.0  20:PEP3 20:PEP4 5.0 1.0  21:PEP3 21:PEP4 5.0 1.0  22:PEP3 22:PEP4 5.0 1.0  1:PEP4 1:PEP5 5.0 1.0  2:PEP4 2:PEP5 5.0 1.0  3:PEP4 3:PEP5 5.0 1.0  4:PEP4 4:PEP5 5.0 1.0  5:PEP4 5:PEP5 5.0 1.0  6:PEP4 6:PEP5 5.0 1.0  7:PEP4 7:PEP5 5.0 1.0  8:PEP4 8:PEP5 5.0 1.0  9:PEP4 9:PEP5 5.0 1.0  10:PEP4 10:PEP5 5.0 1.0  11:PEP4 11:PEP5 5.0 1.0  12:PEP4 12:PEP5 5.0 1.0  13:PEP4 13:PEP5 5.0 1.0  14:PEP4 14:PEP5 5.0 1.0  15:PEP4 15:PEP5 5.0 1.0  16:PEP4 16:PEP5 5.0 1.0  17:PEP4 17:PEP5 5.0 1.0  18:PEP4 18:PEP5 5.0 1.0  19:PEP4 19:PEP5 5.0 1.0  20:PEP4 20:PEP5 5.0 1.0  21:PEP4 21:PEP5 5.0 1.0  22:PEP4 22:PEP5 5.0 1.0 |
| Substrate (seq: KPAEFIRL) docking to pepsin | CABSdock -s 100 -A -C -i 4pep.pdb:A -p KPAEFIRL:EEEEEEEE >& CABS.log | - |
| Substrate (GAETFYVDGA) docking to HIV-1 protease | CABSdock -s 100 -A -C -i 3el1.pdb:AB -f flexibility -p GAETFYVDGA:EEEEEEEEEE --ca-rest-add 52:A 49:A 5.3 1.0 --ca-rest-add 53:A 48:A 4.1 1.0 --ca-rest-add 55:A 46:A 4.6 1.0 --ca-rest-add 52:B 49:B 5.6 1.0 --ca-rest-add 53:B 48:B 4.3 1.0 --ca-rest-add 55:B 46:B 4.2 1.0 >& CABS.log | Protein fragments without internal restraints, file flexibility:  44:A - 57:A 0  44:B - 57:B 0 |

Detailed description of the CABS-dock standalone commands can be found in the wiki pages of the CABS-dock online repository at <http://bitbucket.org/lcbio/cabsdock>.
