# Supplementary material for: Integrative modeling of diverse protein-peptide systems using CABS-dock
Source: PLoS Comput Biol. 2023 Jul 5;19(7):e1011275. doi: 10.1371/journal.pcbi.1011275 (PMC10351741; doi:10.1371/journal.pcbi.1011275)
Supplement: S2 Table — (DOCX) [file pcbi.1011275.s002.docx]

**S2 Table**. **Description of enzyme-substrate systems analyzed in this study**

| **N.o.** | **Enzyme name** | **Enzyme structure PDB ID** | **Substrate sequence** (“--” **cleavage site)** | **Number of docking simulations** | **Predicted models** |
| --- | --- | --- | --- | --- | --- |
| 1 | Pepsin | 4PEP | KPAEF--IRL | 100 | 1000 |
| 2 | Pepsin | 4PEP | HPHLSF--MAI | 100 | 1000 |
| 3 | Pepsin | 4PEP | AFPLEF--IREL | 100 | 1000 |
| 4 | Pepsin | 4PEP | ENESAEAFPLEF--IRELEGER | 100 | 1000 |
| 5 | Pepsin | 4PEP | TMARHPHPHLSF--MAIPPKKNQ | 100 | 1000 |
| 6 | Renin | 3K1W | PFHL--LVYS | 100 | 1000 |
| 7 | Renin | 3K1W | PYIL--KRGS | 100 | 1000 |
| 8 | Renin | 3K1W | DRVYIHPFHLVI--HNESTC | 100 | 1000 |
| 9 | HIV-1 protease | 3EL1 | GAETF--YVDGA | 500 | 5000 |
| 10 | HIV-1 protease | 3EL1 | IRKIL--FLDGI | 500 | 5000 |
| 11 | HIV-1 protease | 3EL1 | TEPISGAETF--YVDGA | 500 | 5000 |
| 12 | HIV-1 protease | 3EL1 | GAETF--YVDGAANRET | 500 | 5000 |
| 13 | HIV-1 protease | 3EL1 | AAGAVASYDY--LVIGGG | 500 | 5000 |
| 14 | HIV-1 protease | 3EL1 | SYFNLNPFEVL--QIDPE | 500 | 5000 |
| 15 | HIV-1 protease | 3EL1 | NVVNSGGMVM--MVPGAG | 500 | 5000 |
