# Supplementary material for: Integrative modeling of diverse protein-peptide systems using CABS-dock
Source: PLoS Comput Biol. 2023 Jul 5;19(7):e1011275. doi: 10.1371/journal.pcbi.1011275 (PMC10351741; doi:10.1371/journal.pcbi.1011275)
Supplement: S3 Table — The average RMSD values were calculated as the arithmetic mean of each set, while the errors were defined as the standard deviations. The first set of models consisted of the 1,000 structures with the lowest interaction energy (or lowest pcaRMSD values), while the second set consisted of the remaining 39,000 models. (DOCX) [file pcbi.1011275.s003.docx]

**S3 Table.** **Average RMSD values for the top 1000 models with the lowest energy/pcaRMSD values and for the remaining set of models**. The average RMSD values were calculated as the arithmetic mean of each set, while the errors were defined as the standard deviations. The first set of models consisted of the 1000 structures with the lowest interaction energy (or lowest pcaRMSD values), while the second set consisted of the remaining 39000 models.

| PDB ID | Set I:  Average RMSD [Å] for 1000 models with the lowest interaction energy | Set II:  Average RMSD [Å] for the remaining models | Set I:  Average RMSD [Å] for 1000 models with the lowest pcaRMSD | Set II  Average RMSD [Å] for the remaining models |
| --- | --- | --- | --- | --- |
| 2E8D | 3.68 ± 0.68 | 8.21 ± 3.79 | 3.64 ± 0.82 | 8.21 ± 3.79 |
| 2NNT | 3.56 ± 0.36 | 9.38 ± 5.51 | 3.45 ± 0.58 | 9.38 ± 5.51 |
| 6TI5 | 4.46 ± 0.87 | 8.43 ± 5.22 | 4.01 ± 0.40 | 8.43 ± 5.22 |
| 6ZRQ | 7.83 ± 3.12 | 11.06 ± 3.18 | 8.44 ± 3.74 | 11.06 ± 3.18 |
| 7Q66 | 6.06 ± 1.18 | 10.51 ± 3.82 | 6.39 ± 3.75 | 10.51 ± 3.81 |
| 7YAT | 5.63 ± 0.41 | 7.08 ± 2.18 | 5.12 ± 0.65 | 7.08 ± 2.18 |
