# Supplementary material for: Integrative modeling of diverse protein-peptide systems using CABS-dock
Source: PLoS Comput Biol. 2023 Jul 5;19(7):e1011275. doi: 10.1371/journal.pcbi.1011275 (PMC10351741; doi:10.1371/journal.pcbi.1011275)
Supplement: S1 Fig — Plots show the correlation between interaction energy values (panel A) and the pcaRMSD parameter (panel B) and the RMSD values calculated using the experimental structures as a reference for a large number (~40,000) of generated protofilament models. At the bottom of each panel there is a color bar describing the colors used for the different values of estimated interaction energy and the pcaRMSD parameter. (DOCX) [file pcbi.1011275.s004.docx]

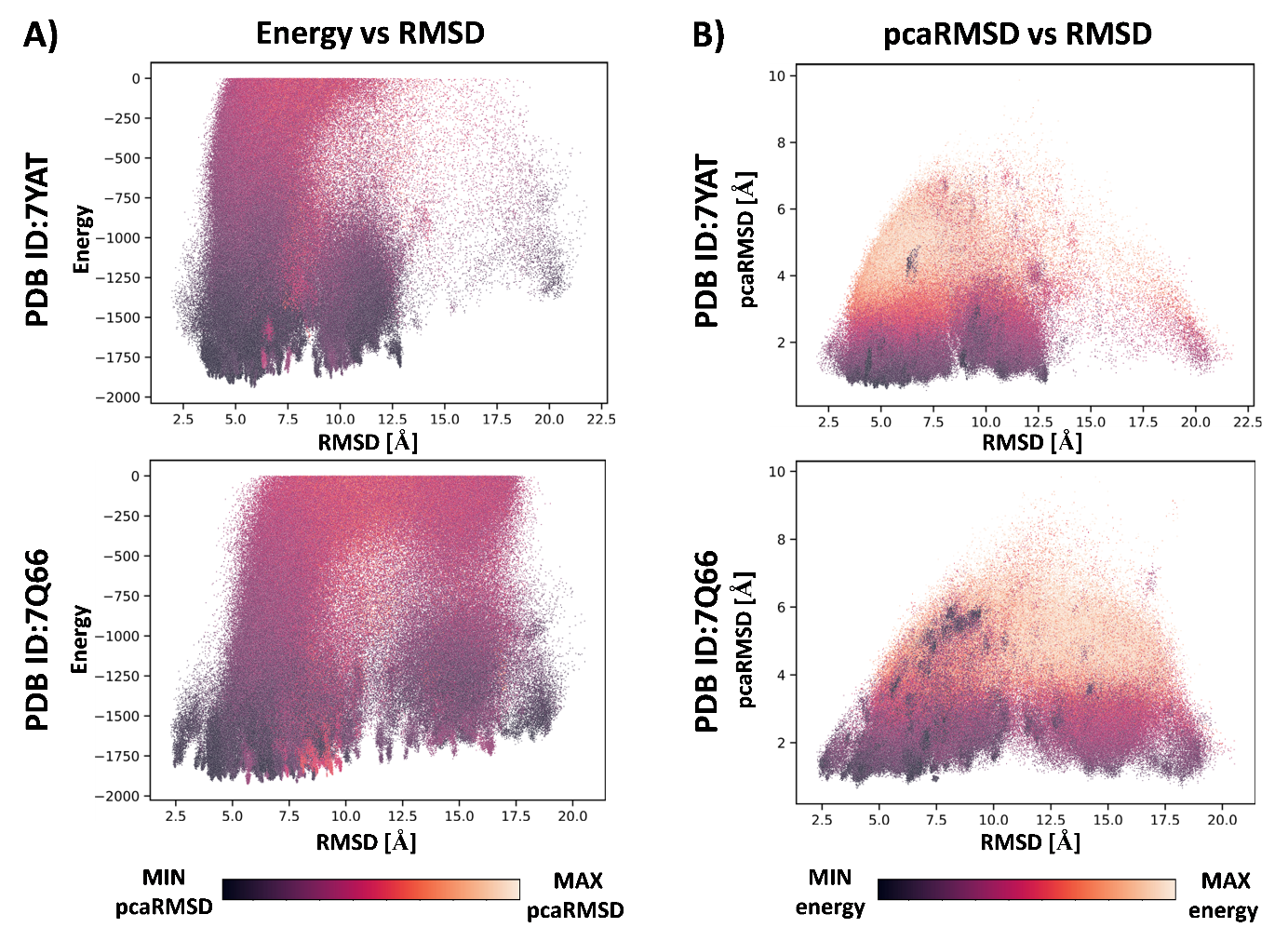


**S1 Fig.** **Correlation between interaction energy, pcaRMSD and RMSD for the two predicted protofilament models.** Plots show the correlation between interaction energy values (panel A) and the pcaRMSD parameter (panel B) and the RMSD values calculated using the experimental structures as a reference for a large number (~40,000) of generated protofilament models. At the bottom of each panel there is a color bar describing the colors used for the different values of estimated interaction energy and the pcaRMSD parameter.
