# Supplementary material for: Integrative modeling of diverse protein-peptide systems using CABS-dock
Source: PLoS Comput Biol. 2023 Jul 5;19(7):e1011275. doi: 10.1371/journal.pcbi.1011275 (PMC10351741; doi:10.1371/journal.pcbi.1011275)
Supplement: S2 Fig — The position of the cleavage site was identified using histogram analysis of the contacts between the carbonyl oxygen atoms of the substrate peptide bonds and the enzyme’s active site. Bar plots show histograms for the cutoff distance (dn ≤ 7 Å). (DOCX) [file pcbi.1011275.s005.docx]

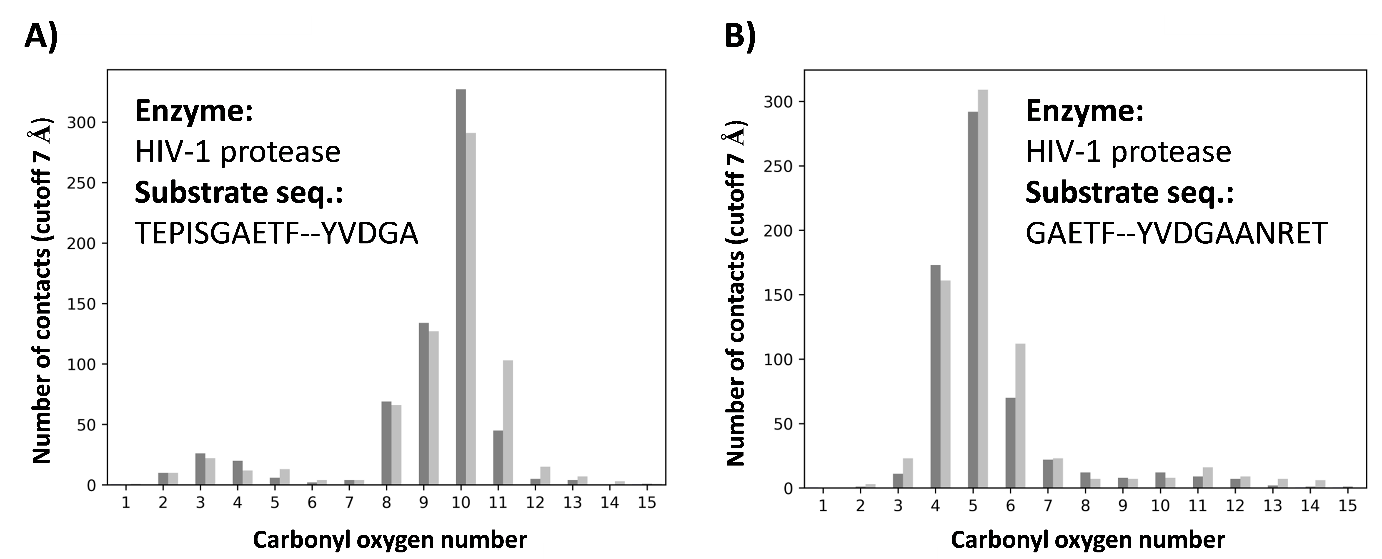


**S2 Fig**. **Results of cleavage site prediction for two HIV-1 protease peptide substrates.** The position of the cleavage site was identified using histogram analysis of the contacts between the carbonyl oxygen atoms of the substrate peptide bonds and the enzyme’s active site. Bar plots show histograms for the cutoff distance (d_n_ ≤ 7 Å).
